# Exploring the Relationship of Paediatric Nutritional Status with Diarrhoeal Disease in Children Below Two Years of Age †

**DOI:** 10.3390/children11111374

**Published:** 2024-11-12

**Authors:** Asif Khaliq, Smita Nambiar-Mann, Yvette D. Miller, Darren Wraith

**Affiliations:** 1 School of Public Health & Social Work, Queensland University of Technology, Brisbane 4059, Australia; yvette.miller@qut.edu.au (Y.D.M.); d.wraith@qut.edu.au (D.W.); 2 School of Exercise & Nutrition Sciences, Queensland University of Technology, Brisbane 4059, Australia; smita.nambiar@qut.edu.au

**Keywords:** malnutrition, coexisting form, paediatrics, diarrhoeal, children

## Abstract

*Background and objective:* Paediatric malnutrition has a synergistic relationship with diarrhoea. In children under two years of age, diarrhoea occurs in more than half of malnutrition cases and is associated with increased duration of illness, increased length of hospital stays, increased morbidity, and mortality. A well-established relationship exists between diarrhoeal and various standalone forms of malnutrition, but their association with coexisting forms of malnutrition (CFM) has not yet been investigated. Thus, this study assessed the association of CFM with diarrhoea among Pakistani children using datasets retrieved from Demographic Health & Survey and UNICEF. *Study design:* A pooled analysis of datasets of Pakistan Demographic & Health Surveys (PDHS) and Multiple Indicator Cluster Surveys (MICS) from the year 2010 to 2018 was conducted. *Methods:* Data of 70,723 children aged below two years were analysed after excluding those with incomplete anthropometry and outliers. *Findings:* Of the total study population, this study reported the presence of either diarrhoea or malnutrition or both in over half of Pakistani children aged between 0 and 23.9 months. Both standalone forms of undernutrition and coexisting forms of undernutrition were significantly associated with increased odds of diarrhoea by 1.07 (1.02 to 1.12) and 1.21 (1.16 to 1.27) times. The practice of breastfeeding, secondary/higher level of maternal education, and improvement in socioeconomic status reduced the risk of diarrhoea, while the children aged between 6 and 23.9 months residing in urban areas showed a high risk of diarrhoea. *Conclusions:* The presence of any type of undernutrition, i.e., standalone and/or coexisting forms were associated with increased risk of diarrhoeal diseases in children. An improvement in socioeconomic status, adherence to the continuation of breastfeeding, and maternal education are keys to reducing the burden of diarrhoea among children.

## 1. Introduction

Children under the age of five years are highly vulnerable to various forms of infections and malnutrition [1]. In 2020, global data from UNICEF, the World Bank, and WHO indicated that approximately 144 million children under five were stunted, 44 million suffered from wasting, and 38.3 million were overweight [2,3]. Malnutrition in children increases the risk, severity, and incidence of infections, including diarrhoeal diseases [4,5]. However, paediatric malnutrition in children, if corrected during the first 1000 days of life, offers a great opportunity for combatting various forms of paediatric infections, including diarrhoea [5,6,7].

Paediatric malnutrition has a well-established and synergistic relationship with diarrhoea [8,9]. For instance, the presence of malnutrition in children amplifies the risk of diarrhoea, while diarrhoea, in turn, leads to malnutrition, i.e., wasting, stunting, and underweight [9,10]. In young children, the coexistence of diarrhoeal illnesses with malnutrition accounts for more than half of malnutrition cases and is associated with increased duration of illness, increased length of stay, increased morbidity, and mortality [11,12,13,14]. Moreover, the coexistence of diarrhoea among malnourished children increased the risk of death by 1.5 (1.1 to 2.2), which increased further to 3.1 (2.3 to 4.2) in children experiencing dehydration compared to those without dehydration [14].

Studies conducted in the past have supported a well-established relationship between diarrhoea with various types of standalone forms of malnutrition, but their association with coexisting forms of malnutrition (CFM) has not yet been investigated [15,16]. The CFM represents simultaneous occurrence of more than one form of nutritional disorder irrespective of age, gender, and health condition [17,18]. Previous studies reported CFM prevalence as ranging from 15.4% to 34.6% among children aged under five years in Pakistan [18,19,20,21].

The primary aim of this study was to examine the relationship of CFM with diarrhoeal disease among the neonates, infants, and young children of Pakistan. Many national and regional reports have consistently highlighted the high endemicity of various types of paediatric illnesses in Pakistan, including the coexistence of diarrhoeal illnesses with paediatric malnutrition [22,23,24]. However, the specific relationship of CFM with diarrhoeal and enteric illnesses is not yet investigated. Based on the existing literature, we hypothesised that children experiencing CFM are also at high risk of developing diarrhoeal diseases. To investigate this hypothesis, data of Pakistan Demographic & Health Survey (PDHS) of 2012–2013 and 2017–2018 and Multiple Indicator Cluster Surveys (MICS) of 2010–2011 (MICS-4), 2014–2017 (MICS-5), and 2017–2020 (MICS-6) were analysed. The findings of this study will provide valuable insights into the severity of the relationship between CFM and diarrhoeal diseases and inform public health interventions.

## 2. Methodology

### 2.1. Study Data and Sampling

In this study, secondary data analysis of PDHS and MICS was performed to assess the associations between CFM and diarrhoeal diseases in children under two years of age. This study used a total of ten different datasets, of which two were PDHS (2012–2013 PDHS and 2017–2018 PDHS), while the remaining eight represented MICS datasets conducted in 2010–2011 (MICS-4), 2014–2017 (MICS-5), and 2017–2020 (MICS-6). These datasets were retrieved from the Demographic & Health Surveys (DHS) and UNICEF data repositories following formal registration and approval of the project.

In each PDHS and MICS, the data were collected from women of reproductive age (15 to 49 years), selected using a multistage stratified cluster sampling technique [22]. In this study, data of all children aged between 0 and 23.9 months with complete anthropometry were included, while those with anthropometric outliers were excluded. The anthropometric outlier values are based on the z-score values defined by the World Health Organization. The z-score value of ±6.00 S.D. for Length/Height for Age (LAZ/HAZ), ±5.00 S.D. for Weight for Height (WHZ), and −6.00 S.D. and +5.00 S.D. for Weight for Age (WAZ) indicate anthropometric outliers [25,26]. Thus, in this study, after excluding anthropometric outlier values, data of 70,723 children were analysed.

### 2.2. Measurement of Study Outcome

In this study, the relationship of various types of malnutrition, including CFM, with diarrhoeal disease was measured among children aged below two years. In each PDHS and MICS, the data collectors did not assess the medical condition of each child, rather they relied on the information provided by the child’s parents/caregiver for assessing any illness. While asking questions related to the prevalence of diarrhoea among children, the interviewer explained the definition of diarrhoea to the respondents, stating that it is characterised by the presence of at least three loose, watery stools within 24 h [27]. A 14-day cut-off period was used for reducing recall biasness of the parents/caregiver. Based on the responses of parents/caregivers, the team of data collectors received two types of health outcomes: normal and diarrhoea. Children with a negative history of diarrhoea during the day and 14 days before data collection were classified as normal, whereas children with complaint of loose, watery stool and/or blood in stool and/or mucus in stool with or without any febrile and/or respiratory condition were classified as diarrhoeal children [28,29].

### 2.3. Measurement of Study Predictor

In each survey, a team of anthropometrists first measured the weight (in kilogram) of each child using SECA 878 U digital scale. For weighing each child, the team took off all the child’s unnecessary clothing, including the child’s shoes, socks, hair ornaments, and jewellery. Due to sociocultural and religious influence, some parents/caregivers objected to the child’s undressing. Therefore, the team weighed those children in a wrap blanket after taking off their unnecessary clothing. In addition to child weight, two anthropometrists (a measurer and an assistant) measured the length/height (in centimetre) using a measuring board. The measurer was involved in taking the measurement, while the assistant was involved in recording the measurement [28]. Anthropometrists involved in weight and length/height measurement received three to four weeks of anthropometry training before the project implementation. For assessing the nutritional status of each child, z-scores were calculated. In this research, we calculated the z-score of each child using WHO AnthroCal^®^ Software. Information related to child length/height, weight, measurement position, sex, age (in months), and participant identity number were imported into the WHO AnthroCal^®^ software for calculating the z-scores. In this study, we considered Weight for Length/Height (WFL/WHZ), Weight for Age (WFA), and Length/Height for Age (LFA/HFA) for assessing nutritional status. We excluded BMI for Age (BAZ) because it overestimates overweight/obesity in children below five years of age [30]. Based on different anthropometric indices, we classified children as normal, wasted, stunted, underweight, and overweight/obese. A child is said to be wasted, stunted, or underweight if the z-score value for WHZ, HAZ, and WAZ falls below −2.00 S.D., respectively. A WHZ value of over +2.00 S.D. indicates the presence of overweight/obesity.

Following categorization of the z-score for each anthropometric index, a new variable derived from HAZ, WAZ, and WHZ was created, and this new variable has nine categories, of which only one category represents a normal healthy child, while the rest of the eight represent a malnourished child. The eight categories of malnutrition were wasting, stunting, underweight, overweight/obesity, coexistence of underweight with wasting, coexistence of underweight with stunting, coexistence of underweight with both wasting and stunting, and coexistence of stunting with overweight/obesity. Furthermore, from these eight categories, four new categories of nutritional status were created: standalone forms of undernutrition, coexisting forms of undernutrition, overnutrition, and nutritional paradox. These are defined as:

*Standalone forms of undernutrition:* presence of a z-score value below −2.00 S.D. for any one anthropometric index.

*Coexisting forms of undernutrition:* presence of a z-score value for any two or more anthropometric indices below −2.00 S.D. The coexisting forms of undernutrition have three types of coexistence of underweight with wasting (having WAZ and WHZ values of ≤−2.00 S.D.), coexistence of underweight with stunting (having WAZ and LAZ/HAZ values of ≤−2.00 S.D.), and coexistence of underweight with both wasting and stunting (having WAZ, WHZ, and LAZ/HAZ values of ≤−2.00 S.D.).

*Overnutrition:* a child whose WHZ z-score value is greater than +2.00 S.D.

*Nutritional paradox:* the presence of overweight/obesity among stunted children reflects paradox forms of malnutrition, and these children have WHZ > +2.00 S.D. and HAZ < −2.00 S.D.

**Figure 1:**
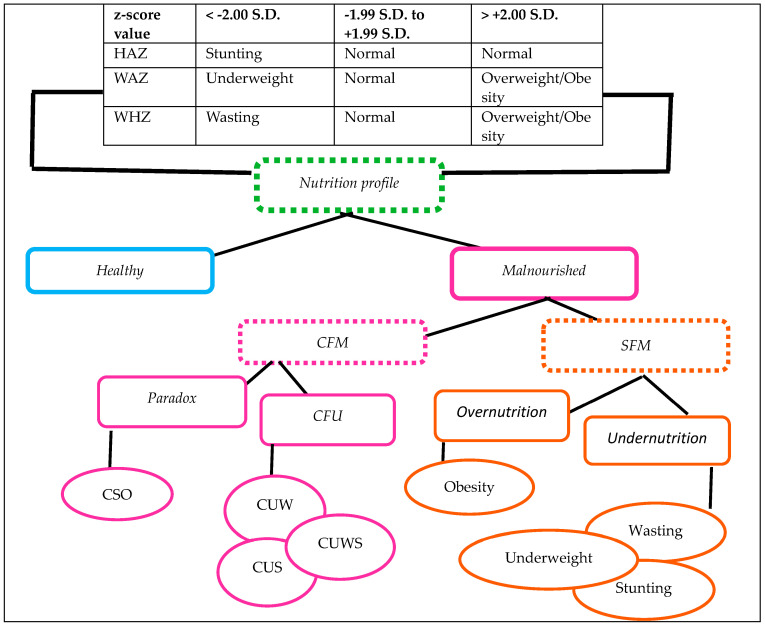
Nutrition profile of children aged between 0 and 23.9 months derived using z-score.

The coexistence of undernutrition and paradox forms of malnutrition were collectively termed as coexisting forms of malnutrition [18,19,20,21]. Additionally, all the categories of different types of malnutrition were merged and showed overall malnutrition. Further details regarding the nutritional profile derived from the z-score of different anthropometric indices are described in Figure 1.

In the figure, CFM = coexisting forms of malnutrition, SFM = standalone forms of malnutrition, CFU = coexisting forms of undernutrition, CSO = coexistence of stunting with overweight/obesity (HAZ ≤ −2.00 S.D. but WHZ ≥ +2.00 S.D.), CUW = coexistence of underweight with wasting (WHZ and WAZ ≤ −2.00 S.D. but HAZ ≠ −2.00 S.D.), CUS = coexistence of underweight with stunting (HAZ and WAZ ≤ −2.00 S.D. but WHZ ≠ −2.00 S.D.), and CUWS = coexistence of underweight with both wasting and stunting (HAZ, WAZ, and WHZ ≤ −2.00 S.D.).

### 2.4. Study Covariates

**Figure 2:**
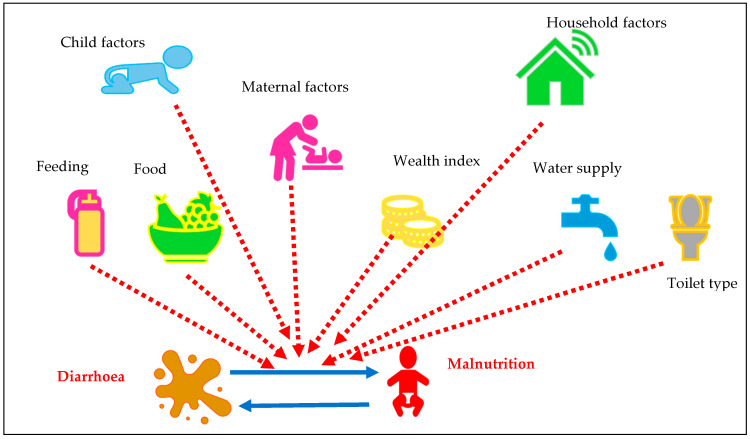
Theoretical relationship of paediatric diarrhoea and paediatric malnutrition with various child, maternal, and household factors.

Covariates for the analyses were assessed in three major categories: child factors, maternal factors, and household and community factors. Child factors included child age, sex, breastfeeding status, and nutritional status. Maternal factors included maternal education, while household and community factors included socioeconomic status and type of place of residence. The relationship of various covariates with the study exposure and outcome variable can be illustrated in Figure 2.

### 2.5. Statistical Analysis

The datasets used in this study were initially screened for each variable. Following data screening, data of all ineligible participants were removed sequentially from the datasets. Later, descriptive data analysis was performed for each variable, in which we calculated frequency and percentages for categorical variables.

Inferentially, data modelling was performed using unordered binomial logistic regression. The reference category chosen was a normal healthy child for both study outcome and study predictor. However, for each study covariate, the reference category is presented in Table 1. The association of study outcome with the study predictor variable and related covariates was measured separately. A *p*-value of 0.05 and less and confidence interval of 95% was considered significant. Moreover, the relationship between predictor variable and all covariates with child health status (outcome variable) was assessed together using a backward elimination method. Multicollinearity was also examined, but we did not find multicollinearity between any covariate. Certain covariates having *p*-value > 0.05 were removed sequentially from the model, with nutritional status retained in the model regardless of its *p*-value.

### 2.6. Ethical Consideration

Access to the Pakistan Demographic and Health Survey (PDHS) data was granted by the data archivist of the Demographic and Health Surveys (DHS) data repository following the formal registration of study objectives. A formal approval letter for data access and use was provided by the DHS data archivist. Similarly, the Multiple Indicator Cluster Survey (MICS) data were accessed through the UNICEF data repository upon formal registration on the UNICEF MICS website, with approval granted by the UNICEF data archivist via email (See Supplementary File S1). Both datasets were provided in a deidentified format, excluding any participant identifiers, such as names, phone numbers, national identity numbers, or addresses, thus maintaining participant confidentiality and privacy.

As this secondary analysis used deidentified data, informed consent was not applicable. However, to ensure compliance with ethical standards, a formal ethics application was submitted to and approved by the Queensland University of Technology Human Research Ethics Committee (UHREC), under approval number 2000-000177. This study adhered to ethical standards and good clinical practices in secondary data analysis, following guidelines for confidentiality and responsible data use as stipulated by the DHS and UNICEF.

## 3. Results

The characteristics of the samples are shown in Table 1.

Overall, 44.6% of children were healthy and 55.4% had either diarrhoea (12.6%), malnutrition (31.9%), or diarrhoea-related malnutrition (10.9%). Among malnourished children, 24.7% had CFM, of which 23.2% had coexisting forms of undernutrition, while 1.5% had a nutritional paradox. Coexistence of underweight with stunting was the most highly prevalent type of CFM, followed by underweight with stunting and underweight with both wasting and stunting (see Table 1).

### Association of Paediatric Diarrhoea with Various Forms of Malnutrition

Compared to a normal healthy child, the presence of malnutrition in children increases the odds of diarrhoea to 1.13 (1.09 to 1.18) fold. The undernutrition of both types (standalone and coexisting forms of undernutrition) was associated with paediatric diarrhoea. However, paediatric overnutrition (overweight/obesity) and the nutritional paradox were not associated with diarrhoea. The odds of paediatric diarrhoea were significantly higher in children aged 6 to 23.9 months compared to those under 6 months. Similarly, children living in urban areas had 1.16 times (1.11 to 1.21) higher odds of diarrhoea than those in rural areas of Pakistan. An improvement in socioeconomic status was associated with a 9% to 42% decrease in the odds of diarrhoea. Additionally, significantly lower odds of diarrhoea were observed among non-breastfed children and children of mothers with higher education compared to breastfed children and children of uneducated mothers (Table 2).

Among various forms of CFM, the coexistence of underweight with wasting, underweight with stunting, and underweight with both wasting and stunting were associated with an increase in the odds of paediatric diarrhoea by 1.20 (1.12 to 1.29), 1.20 (1.13 to 1.27), and 1.26 (1.17 to 1.36), respectively, compared to a healthy weight child. However, no association between the nutritional paradox and paediatric diarrhoea was reported. Among other factors, higher odds of diarrhoea were observed in children aged between 6 months and 23.9 months and those living in urban areas. Conversely, lower odds of diarrhoea were found among female children, non-breastfed children, children with educated mothers, and children from poorer to richest families compared to male children, breastfed children, children with uneducated mothers, and children from the poorest families (Table 3).

The relationship between various forms of coexisting undernutrition (underweight with wasting, underweight with stunting, and underweight with both wasting and stunting) and paediatric diarrhoea showed no significant association when compared to underweight children (Table 4).

The odds of diarrheal disease were significantly higher among children living in urban areas. Similarly, children aged 6 to 23.9 months had greater odds of diarrhoea compared to those under six months. However, abstinence of breastfeeding practices, maternal education at the secondary or higher level, and improvements in socioeconomic status were all significantly associated with lower odds of diarrheal disease (Table 4).

## 4. Discussion

This study examined the relationship of all forms of paediatric malnutrition (undernutrition, overnutrition, coexisting forms of undernutrition, and nutritional paradox) with diarrhoeal disease among Pakistani children aged between 0 and 23.9 months. Children experiencing standalone forms of malnutrition showed 1.07 (1.02 to 1.12) higher odds of diarrhoea, while the odds of diarrhoea increased to 1.21 (1.16 to 1.27) in children suffering from coexisting forms of undernutrition. Similarly, different studies and reviews conducted in the past reported a significant association between paediatric undernutrition and diarrhoea [7,31,32,33]. However, with other forms of malnutrition (i.e., overnutrition and nutritional paradox), no association with paediatric diarrhoea was seen. Thus, the presence of paediatric undernutrition, specifically coexisting forms of undernutrition, is associated with an increase in the odds of diarrhoea in children below two years of age.

Of the total study population, this study reported the presence of either diarrhoea or malnutrition or both in over half of Pakistani children aged between 0 and 23.9 months. The findings of this study depicted undernutrition as a highly prevalent form of malnutrition in children below two years. Similarly, studies conducted in China, India, and Pakistan also acknowledged a high prevalence of paediatric undernutrition, compared with paediatric overnutrition and/or nutritional paradox [28,34,35]. The national nutrition surveys, reports of demographics health and surveys, and a global nutrition report also indicated high prevalence of paediatric undernutrition in most of the Asian and African countries compared to paediatric overnutrition and/or nutritional paradox [36,37]. Furthermore, this study also depicted a high prevalence of CFM, including coexisting forms of undernutrition compared with standalone forms of malnutrition/undernutrition in children aged below two years. Similarly, our previous study conducted in children aged below five years showed higher coexisting forms of undernutrition compared with standalone forms of undernutrition [18]. Besides paediatric malnutrition, our study reported diarrhoea prevalence in 23.5% of children, of which 10.9% had malnutrition (co-occurrence of malnutrition with diarrhoea). Diarrhoea in children is the second leading cause of death after pneumonia. Compared to a normal well-nourished child with diarrhoea, concomitant existence of diarrhoea with undernutrition increases the death propensity by 1.6 times in children [38]. Other studies and reviews also showed a high risk of diarrhoea-associated morbidity and/or diarrhoea-associated mortality among undernourished children, compared with well-nourished/healthy children [6,39,40,41]. The concomitant existence of diarrhoea with undernutrition can be averted by adopting four diarrhoea case management measures: fluid and electrolytes replacement by prompt provision of Oral Rehydration Solution (ORS), use of zinc sulphate for reducing the frequency and duration of diarrhoeal episodes, use of probiotics for better immunity, and continuous provision of adequate and healthy diet for supply of calories and energy [39,42,43].

Our study reported higher odds of diarrhoea in children aged between 6 and 23.9 months, compared to young children aged below six months. Other studies have also identified that preschool children are more vulnerable to developing various types of preventable illnesses, including diarrhoea [44,45]. The high incidence of various types of preventable illnesses, including diarrhoea, among children aged between 6 and 23.9 months might be due to their contact movement (crawling), early cessation of breastmilk, early initiation of weaning, and partially developed immune system [43]. Additionally, the findings of this study reported a protective role of breastfeeding with diarrhoea. The practice of continuation of breastfeeding in children under two years reduced the odds of diarrhoea in children below two years of age by 0.80 (0.76 to 0.84). Similarly, different studies conducted in Ethiopia, Saudi Arabia, and Tanzania have found a protective effect of breastfeeding with diarrhoea [46,47,48]. This was further supported by a systematic review, which also demonstrated the protective effect of breastfeeding with diarrhoea [42]. However, the risk of diarrhoea increased to over 10-fold in non-breastfed children [42,46]. In this regard, educating mothers and caregivers about the beneficial effect of the continuation of breastfeeding and other feeding indicators proposed in the Infants & Young Child Feeding (IYCF) guidelines can help to avert both malnutrition and diarrhoea in children below two years of age [47,49,50]. Many studies conducted in the past also supported maternal education for enhancing healthcare and better quality of life [51,52]. Similarly, our study reported lower odds of paediatric diarrhoea in children of mothers having either secondary or higher level of education, compared to non-educated mothers. Similarly, other studies also supported that deprivation of maternal education to a secondary/higher level is a basic cause for high episodes of diarrhoea in children below two years of age [43,53,54]. However, a study by Khaliq, et al. (2022) depicted that maternal knowledge and education is not solely responsible for paediatric diarrhoea; rather, a lack of appropriate preventive measures due to environmental and behavioural barriers mainly contribute to paediatric diarrhoea [43]. Moreover, children living in urban residence showed higher odds of paediatric diarrhoea compared with children of rural areas. Conversely, many studies supported that urbanization is associated with better health due to the provision of safe drinking water and improved sanitation facilities [43,46,55,56,57]. Hence, it is not clear why urbanisation is associated with higher odds of paediatric diarrhoea. Further research which could specifically assess the relationship of each housing and sanitation indicator with diarrhoea can better examine the relationship of diarrhoea with urbanisation and associated factors.

### Strengths and Limitations

Our study is the first study to examine the relationship of CFM with diarrhoea in children under five years of age. The information related to health and nutrition status of children was collected by a team of trained and experienced data collectors. The data collectors assessed the nutrition status using calibrated and standardised devices after following the standard operating procedure guided by the WHO for anthropometric measurement [58]. Similarly, the data related to medical illness were collected using the Integrated Management & Childhood Illness (IMCI) guidelines [28,29]. Thus, in our study, the health and nutrition status of children were measured using standardised guidelines.

On the other hand, we need to acknowledge certain limitations of this study, which include *(1) Cross-sectional study design:* the cross-sectional study design is a major limitation of this study, which has reduced the internal validity and reliability of this study. *(2) Recall biasness:* the parents in this study were asked to report last two-week medical history of common preventable illnesses of their children, and there might be chances that the children experienced illness of our interest over two-week periods. *(3) Lack of severity and chronicity assessment:* our study also overlooked the severity and chronicity of paediatric health and nutrition disorders. For example, our study did not assess the severity of various types of malnutrition, i.e., moderate malnutrition and severe malnutrition. Similarly, the degree of dehydration associated with diarrhoea illnesses was not assessed as well. Owing to these reasons, we need to consider the limitations of our study, when using the findings of this study for devising a policy against the prevention of CFM among children of diarrhoeal and enteric illnesses.

## 5. Conclusions

Our study provides a snapshot about the relationship of diarrhoeal and enteric illnesses with the various forms of malnutrition. We found malnutrition, and/or paediatric diarrhoea and/or its coexistence in more than half of children of Pakistan. In general, undernutrition of both types, standalone forms of undernutrition and coexisting forms of undernutrition, were associated with diarrhoeal diseases in children. Tackling a high burden of diarrhoeal diseases with undernutrition requires knowledge and understanding about the synergistic and bidirectional relationship of diarrhoeal diseases with various forms of undernutrition. Previous studies have reported that adequate nutrition and adherence to infant feeding practices serves to protect children from malnutrition, which, in turn, protects them from many paediatric illnesses, including diarrhoea. For better understanding the relationship between paediatric CFM and diarrhoeal disease, there is a need to carry out longitudinal studies, which could assess the duration, severity, and associated symptoms among children with diarrhoea.

## Figures and Tables

**Table 1:** Characteristics of study sample.

Variable	Category	Total (*N* = 70,723)
*Health and Nutrition status*		
Overall health status	Diarrhoea	8907 (12.6%)
	Malnourished	22,591 (31.9%)
	Malnourished with diarrhoea.	7688 (10.9%)
	Healthy	31,537 (44.6%)
Presence of diarrhoea	Yes	16,595 (23.5%)
	No	54,128 (76.5%)
Nutrition status	Normal	40,444 (57.2%)
	Malnourished	30,279 (42.8%)
	Standalone forms of malnutrition	- 12,822 (18.2%)
	○ Undernutrition *	○ 12,138 (17.2%)
	○ Overnutrition ^¥^	684 (1.0%)
	
	Coexisting forms of malnutrition ^€^	- 17,457 (24.7%)
	○ Coexistence of undernutrition ^α^	○ 16,428 (23.2%)
	▪ CUW	▪ 4572 (6.5%)
	▪ CUS	▪ 7773 (11.0%)
	▪ CUWS	▪ 4083 (5.8%)
	
	Nutritional Paradox ^β^	- 1029 (1.5%)
*Child factors*		
Sex of child	Male	35,935 (50.8%)
	Female	34,788 (49.2%)
Child age in months	0 to 5.9 months	17,786 (25.1%)
	6 to 11.9 months	18,721 (26.5%)
	12.0 to 17.9 months	15,756 (22.3%)
	18.0 to 23.9 months	18,460 (26.1%)
Use of breastmilk	Yes	53,848 (76.1%)
	No	16,875 (23.9%)
*Maternal factors*		
Maternal education	No education	35,598 (50.3%)
	Primary	18,340 (25.9%)
	Secondary or Higher	16,785 (23.7%)
*Household and community factors*		
Socioeconomic status	Poorest	15,348 (21.7%)
	Poorer	15,031 (21.3%)
	Middle	14,827 (21.0%)
	Richer	13,814 (19.5%)
	Richest	11,703 (16.5%)
Type of place of residence	Rural	46,324 (67.0%)
	Urban	22,827 (33.0%)

Where * = undernutrition including presence of either stunting or wasting or underweight in a child, ^¥^ = presence of overweight/obesity in an individual child, ^€^ = presence of more than one form of nutritional disorder, and this includes combined statistics of coexistence of undernutrition and nutritional paradox, ^α^ = presence of more than one form of undernutrition in an individual, CUW = coexistence of underweight with wasting, CUS = coexistence of underweight with stunting, CUWS = coexistence of underweight with both wasting and stunting, ^β^ = simultaneous presence of stunting with overweight/obesity in an individual.

**Table 2:** Association of paediatric diarrhoea with malnutrition and its various types.

Variable	Categories	Diarrhoea ∞		
		Unadjusted Odds (CI: 95%)	Adjusted Odds (CI: 95%) ^1^	Adjusted Odds (CI: 95%) ^2^
Nutritional status	Normal	Ref	Ref	-
	Malnourished	1.21 (1.16 to 1.24) *	1.13 (1.09 to 1.18) *	
Types of malnutrition	Normal	Ref	-	Ref
	Undernutrition	1.11 (1.05 to 1.16) *		1.07 (1.02 to 1.12) *
	Overnutrition	0.82 (0.67 to 0.99) *		0.90 (0.74 to 1.09)
	CFU	1.32 (1.26 to 1.37) *		1.21 (1.16 to 1.27) *
	Paradox	0.84 (0.72 to 0.99) *		0.85 (0.72 to 1.00)
Sex of child	Male	Ref	-	-
	Female	0.97 (0.94 to 1.00)		
Child age in months	0 to 5.9 months	Ref	Ref	Ref
	6 to 11.9 months	1.51 (1.44 to 1.59) *	1.48 (1.41 to 1.56) *	1.46 (1.39 to 1.54) *
	12 to 17.9 months	1.42 (1.35 to 1.49) *	1.33 (1.27 to 1.41) *	1.32 (1.25 to 1.39) *
	18 to 23.9 months	1.23 (1.17 to 1.30) *	1.10 (1.04 to 1.16) *	1.08 (1.02 to 1.14) *
Breastfeeding status	Yes	Ref	Ref	Ref
	No	0.84 (0.81 to 0.87) *	0.80 (0.76 to 0.84) *	0.80 (0.76 to 0.84) *
Maternal education	No education	Ref	Ref	Ref
	Primary	0.87 (0.84 to 0.91) *	0.97 (0.92 to 1.01)	0.97 (0.93 to 1.01)
	Secondary or Higher	0.69 (0.66 to 0.72) *	0.84 (0.80 to 0.89) *	0.84 (0.80 to 0.89) *
Socioeconomic status	Poorest	Ref	Ref	Ref
	Poorer	0.92 (0.87 to 0.97) *	0.91 (0.86 to 0.96) *	0.91 (0.87 to 0.86) *
	Middle	0.81 (0.76 to 0.85) *	0.80 (0.75 to 0.84) *	0.80 (0.75 to 0.85) *
	Richer	0.71 (0.67 to 0.75) *	0.70 (0.65 to 0.74) *	0.71 (0.66 to 0.75) *
	Richest	0.59 (0.55 to 0.62) *	0.57 (0.53 to 0.62) *	0.58 (0.54 to 0.63) *
Type of place of residence	Rural	Ref	Ref	Ref
	Urban	0.92 (0.88 to 0.95) *	1.16 (1.11 to 1.22) *	1.16 (1.11 to 1.21) *

∞ = The reference category for assessing the determinants of standalone forms of undernutrition and of coexisting forms of undernutrition was a normal healthy child. ^1^ = Association of paediatric diarrhoea with overall malnutrition was assessed after adjusting child age, breastfeeding status, maternal education, socioeconomic status, and place of residence. ^2^ = Association of paediatric diarrhoea with various types of malnutrition was assessed after adjusting child age, breastfeeding status, maternal education, socioeconomic status, and place of residence. * = Significant relationship or the *p*-value ≤ 0.05.

**Table 3:** Association of paediatric diarrhoea with various forms of CFM.

Variable	Categories	Diarrhoea ^£^	
		Unadjusted Odds (CI: 95%)	Adjusted Odds (CI: 95%) ^1^
Coexisting forms of malnutrition	Normal	Ref	Ref
	CUW	1.28 (1.19 to 1.37) *	1.20 (1.12 to 1.29) *
	CUS	1.30 (1.23 to 1.37) *	1.20 (1.13 to 1.27) *
	CUWS	1.41 (1.31 to 1.51) *	1.26 (1.17 to 1.36) *
	CSO	0.84 (0.72 to 0.99) *	0.85 (0.72 to 1.01)
Sex of child	Male	Ref	Ref
	Female	0.95 (0.91 to 0.99) *	0.95 (0.92 to 0.99) *
Child age in months	0 to 5.9 months	Ref	Ref
	6 to 11.9 months	1.53 (1.44 to 1.61) *	1.47 (1.39 to 1.55) *
	12 to 17.9 months	1.44 (1.36 to 1.52) *	1.32 (1.25 to 1.41) *
	18 to 23.9 months	1.24 (1.17 to 1.32) *	1.07 (1.01 to 1.15) *
Breastfeeding status	Yes	Ref	Ref
	No	0.84 (0.81 to 0.88) *	0.80 (0.76 to 0.84) *
Maternal education	No education	Ref	Ref
	Primary	0.88 (0.84 to 0.92) *	0.97 (0.93 to 1.03)
	Secondary or Higher	0.72 (0.66 to 0.73) *	0.85 (0.80 to 0.91) *
Socioeconomic status	Poorest	Ref	Ref
	Poorer	0.90 (0.85 to 0.95) *	0.90 (0.84 to 0.95) *
	Middle	0.81 (0.76 to 0.86) *	0.81 (0.76 to 0.86) *
	Richer	0.71 (0.67 to 0.76) *	0.71 (0.66 to 0.76) *
	Richest	0.59 (0.55 to 0.62) *	0.58 (0.53 to 0.64) *
Type of place of residence	Rural	Ref	Ref
	Urban	0.95 (0.91 to 0.99) *	1.15 (1.10 to 1.21) *

^£^ = The reference category for assessing the association of diarrhoea with CFM was a normal healthy child having z-score value between −1.99 S.D. and +1.99 S.D. ^1^ = Association of paediatric diarrhoea with coexisting forms of malnutrition was assessed after adjusting child age, child sex, breastfeeding status, maternal education, socioeconomic status, and place of residence. * = Significant relationship or the *p*-value ≤ 0.05.

**Table 4:** Association of paediatric diarrhoea with various forms of coexisting forms of undernutrition.

Variable	Categories	Diarrhoea ^£^	
		Unadjusted Odds (CI: 95%)	Adjusted Odds (CI: 95%) ^1^
Coexisting forms of undernutrition	Underweight	Ref	Ref
	CUW	0.92 (0.81 to 1.05)	0.91 (0.79 to 1.03)
	CUS	0.93 (0.82 to 1.05)	0.91 (0.80 to 1.03)
	CUWS	1.01 (0.89 to 1.15)	0.95 (0.83 to 1.09)
Sex of child	Male	Ref	-
	Female	1.00 (0.94 to 1.07)	
Child age in months	0 to 5.9 months	Ref	Ref
	6 to 11.9 months	1.49 (1.34 to 1.67) *	1.49 (1.33 to 1.66) *
	12 to 17.9 months	1.42 (1.28 to 1.59) *	1.37 (1.23 to 1.53) *
	18 to 23.9 months	1.20 (1.07 to 1.33) *	1.09 (0.97 to 1.22)
Breastfeeding status	Yes	Ref	Ref
	No	0.82 (0.76 to 0.88) *	0.77 (0.71 to 0.83) *
Maternal education	No education	Ref	-
	Primary	0.93 (0.86 to 1.01)	
	Secondary or Higher	0.81 (0.74 to 0.89) *	
Socioeconomic status	Poorest	Ref	Ref
	Poorer	0.91 (0.84 to 1.01)	0.88 (0.81 to 0.96) *
	Middle	0.91 (0.83 to 1.00)	0.84 (0.76 to 0.93) *
	Richer	0.75 (0.68 to 0.83) *	0.65 (0.58 to 0.73) *
	Richest	0.65 (0.57 to 0.73) *	0.53 (0.46 to 0.61) *
Type of place of residence	Rural	Ref	Ref
	Urban	0.82 (0.76 to 0.88) *	1.22 (1.12 to 1.33) *

^£^ = The reference category for assessing the association of diarrhoea with CFU was an underweight child having WAZ of less than −2.00 S.D. ^1^ = Association of paediatric diarrhoea with coexisting forms of undernutrition was assessed after adjusting child age, breastfeeding status, socioeconomic status, and place of residence. * = Significant relationship or the *p*-value ≤ 0.05.

## Data Availability

The data from this study can be retrieved from the DHS program (www.dhsprogram.com, accessed on 11 November 2019) and UNICEF (www.mics.unicef.org/surveys, accessed on 29 January 2020).

## Supplementary Materials

The following supporting information can be downloaded at: https://www.mdpi.com/article/10.3390/children11111374/s1, File S1: Reporting checklist for cross sectional study.
